# On Compactness of Products of Toeplitz Operators

**DOI:** 10.1007/s11785-024-01625-y

**Published:** 2024-11-16

**Authors:** Trieu Le, Tomas Miguel Rodriguez, Sönmez Şahutoğlu

**Affiliations:** 1 Department of Mathematics & Statistics, University of Toledo, 2801 W. Bancroft, Toledo, OH 43606 USA; 2 Mathematics and Statistics, Kenyon College, 106 College Park Dr, Gambier, OH 43022 USA

**Keywords:** Toeplitz operator, Compact, Bergman space, Polydisc, Primary 47B35, Secondary 32A36

## Abstract

We study compactness of product of Toeplitz operators with symbols continuous on the closure of the polydisc in terms of behavior of the symbols on the boundary. For certain classes of symbols *f* and *g*, we show that $T_fT_g$ is compact if and only if *fg* vanishes on the boundary. We provide examples to show that for more general symbols, the vanishing of *fg* on the whole polydisc might not imply the compactness of $T_fT_g$. On the other hand, the reverse direction is closely related to the zero product problem for Toeplitz operators on the unit disc, which is still open.

## Introduction

Let $\Omega$ be a bounded domain in $\mathbb {C}^n$. The Bergman space $A^2(\Omega )$ consists of all holomorphic functions on $\Omega$ that are square integrable with respect to the Lebesgue volume measure *dV*. The orthogonal projection $P:L^2(\Omega )\rightarrow A^2(\Omega )$ is known as the Bergman projection. For a bounded measurable function *f* on $\Omega$, the Toeplitz operator $T_{f}: A^2(\Omega )\rightarrow A^2(\Omega )$ is defined as

$$\begin{aligned} T_fh = P(fh) \end{aligned}$$

for $h\in A^2(\Omega )$. We call *f* the symbol of $T_f$.

There is an extensive literature on the study of Toeplitz operators on various domains. In this paper, we are particularly interested in the case the domain is the polydisc and compactness of product of Toeplitz operators whose symbols are continuous up to the boundary.

A classical approach to compactness of Toeplitz operators involves the Berezin transform. For finite sum of finite products of Toeplitz operators on the Bergman space of the unit disc, the Axler–Zheng Theorem [1, Theorem 2.2] characterizes compactness in terms of the behavior of the Berezin transform of the operator. In higher dimensions, the Axler–Zheng Theorem is extended to the case of the polydisc as seen in [2] and [3, p. 232], and the unit ball as shown in [4, Theorem 9.5]. Recently, there have been a few generalizations of this result in different directions. See, for instance, [5–8].

In this paper, we study compactness of products of Toeplitz operators in terms of the behavior of the symbols on the boundary. More specifically, we would like to characterize functions *f*, *g* that are continuous on $\overline{\mathbb {D}^n}$ such that $T_{f}T_{g}$ is compact.

Coburn [9, Lemma 2] showed that on the Bergman space over unit ball $\mathbb {B}$, for *f* a continuous function on $\overline{\mathbb {B}}$, the Toeplitz operator $T_f$ is compact if and only if $f=0$ on $b\mathbb {B}$. Furthermore, [9, Theorem 1] established a $*$-isomorphism $\sigma :\tau (\mathbb {B}) / \mathscr {K} \rightarrow C(b\mathbb {B})$ satisfying

$$\begin{aligned} \sigma (T_{f}+ \mathscr {K}) = f|_{b\mathbb {B}}, \end{aligned}$$

where $\tau (\mathbb {B})$ is the Toeplitz algebra generated by $\{T_{\varphi }: \varphi \in C(\overline{\mathbb {B}})\}$ and $\mathscr {K}$ is the ideal of compact operators on $A^2(\mathbb {B})$. As a consequence, we see that for $f_1,\ldots ,f_N\in C(\overline{\mathbb {B}})$, the product $T_{f_1}\cdots T_{f_N}$ is compact if and only if the product $f_1\cdots f_N = 0$ on $b\mathbb {B}$.

On the polydisc $\mathbb {D}^n$, the first author [10] showed that, in the context of weighted Bergman spaces, for $f\in C(\overline{\mathbb {D}^n})$, the Toeplitz operator $T_f$ is compact if and only if *f* vanishes on $b\mathbb {D}^n$, the (topological) boundary of $\mathbb {D}^n$. Generalizing this result, the second and the third authors in [11] proved that compactness of the Toeplitz operator with a symbol continuous on the closure of a bounded pseudoconvex domain in $\mathbb {C}^n$ with Lipschitz boundary is equivalent to the symbol vanishing on the boundary of the domain.

Motivated by Coburn’s aforementioned result, one may expect that the necessary and sufficient condition for $T_{f}T_{g}$ to be compact is that *fg* vanishes on $b\mathbb {D}^n$. However, we shall see in our results and examples that while the above statement holds for a certain class of symbols, sufficiency is false in general. On the other hand, necessity is closely related with the famous “zero product problem” in the theory of Toeplitz operators on the unit disc, which is still wide open.

## Main Result

Let $T= \sum ^{N}_{j=1} T_{f_{j,1}} \cdots T_{f_{j,m_j}}$ be a finite sum of finite products of Toeplitz operators with $f_{j,k}\in C(\overline{\mathbb {D}})$. Coburn’s aforementioned result implies that compactness of the operator *T* on $A^2(\mathbb {D})$ is equivalent to $\sum ^{N}_{j=1} {f_{j,1}} \cdots {f_{j,m_j}} = 0$ on the circle. Therefore, throughout the paper we will assume that $n\ge 2$ as the case $n=1$ is well understood.

Before we state our results, we define the restriction operator $R_{k,\xi }:C(\overline{\mathbb {D}^n})\rightarrow C(\overline{\mathbb {D}^{n-1}})$ for $\xi \in \mathbb {T}$ and $k=1,\ldots ,n$ as follows.

$$\begin{aligned} R_{1,\xi }f(z_1,\ldots ,z_{n-1})=&\,f(\xi ,z_1,\ldots ,z_{n-1}),\\ R_{n,\xi }f(z_1,\ldots ,z_{n-1})=&\,f(z_1,\ldots ,z_{n-1},\xi ), \end{aligned}$$

and

$$\begin{aligned} R_{k,\xi }f(z_1,\ldots ,z_{n-1})=f(z_1,\ldots ,z_{k-1},\xi ,z_k,\ldots ,z_{n-1}) \end{aligned}$$

for $$2\le k\le n-1$$ and $$f\in C(\overline{\mathbb {D}^n})$$.

In our main result, we give a characterization of compactness of the finite sum of finite products of Toeplitz operators in terms of the vanishing of the operator restricted to the polydiscs in the boundary. We recall that $b\mathbb {D}^n$ consists of all $z=(z_1,\ldots ,z_n)\in \overline{\mathbb {D}^n}$ such that $|z_j|=1$ for some *j*.

### Theorem 1

Let $T= \sum ^{N}_{j=1} T_{f_{j,1}} \cdots T_{f_{j,m_j}}$ be a finite sum of finite products of Toeplitz operators on $A^2(\mathbb {D}^n)$ for $f_{j,k} \in C(\overline{\mathbb {D}^n})$ with $n\ge 2$. Then *T* is compact on $A^2(\mathbb {D}^n)$ if and only if

$$\begin{aligned} \sum ^{N}_{j=1} T_{R_{k,\xi }f_{j,1}} \cdots T_{R_{k,\xi }f_{j,m_j}} = 0 \end{aligned}$$

on $A^2(\mathbb {D}^{n-1})$ for all $\xi \in \mathbb {T}$ and $1\le k\le n$.

As an immediate corollary we get the following.

### Corollary 1

Let $f_j\in C(\overline{\mathbb {D}^n})$ for $1\le j\le m$. Assume that for each $\xi \in \mathbb {T}$ and $1\le k \le n$ there exists *j* such that $R_{k,\xi }f_j=0$ on $\mathbb {D}^{n-1}$. Then $T_{f_m}\cdots T_{f_1}$ is compact on $A^2(\mathbb {D}^n)$.

## Applications

Let $\varphi$ and $\psi$ be two functions in $C(\overline{\mathbb {D}})$. We define $f(z,w) = \varphi (w)$ and $g(z,w) = \psi (w)$ for $z,w\in \overline{\mathbb {D}}$. Then for any $\xi \in \mathbb {T}$,

$$R_{1,\xi }f(w) = f(\xi ,w) = \varphi (w),\quad R_{1,\xi }g(w) = g(\xi ,w)=\psi (w)\quad \text { for } w\in \mathbb {D}$$

and

$$R_{2,\xi }f(z) = \varphi (\xi ),\quad R_{2,\xi }g(z) = \psi (\xi )\quad \text { for } z\in \mathbb {D}.$$

By Theorem 1, the product $T_fT_g$ is a compact operator on $A^2(\mathbb {D}^2)$ if and only if $T_{\varphi }T_{\psi } = 0$ on $A^2(\mathbb {D})$ and $\varphi (\xi )\psi (\xi ) = 0$ for all $\xi \in \mathbb {T}$. Since the second condition is actually a consequence of the first, we conclude that for such *f* and *g*, the product $T_fT_g$ is compact on $A^2(\mathbb {D}^2)$ if and only if $T_{\varphi }T_{\psi }= 0$ on $A^2(\mathbb {D})$, which is equivalent to $T_{f}T_{g} = 0$ on $A^2(\mathbb {D}^2)$.

### Example 1

Let

$$\varphi (w) = {\left\{ \begin{array}{ll} 1-2|w| &  \text { for } 0\le |w|\le \frac{1}{2}\\ 0 &  \text { for } |w|>\frac{1}{2}, \end{array}\right. }$$

and

$$\psi (w) = {\left\{ \begin{array}{ll} 0 &  \text { for } 0\le |w|\le \frac{1}{2}\\ 2|w|-1 &  \text { for } |w|>\frac{1}{2}. \end{array}\right. }$$

Using polar coordinates, one can check that both operators $T_{\varphi }$ and $T_{\psi }$ are diagonalizable with respect to the standard orthonormal basis and their eigenvalues are all strictly positive. Hence $T_{\varphi }T_{\psi }\not \equiv 0$ on $A^2(\mathbb {D})$. On the other hand, $\varphi \psi = 0$ on $\overline{\mathbb {D}}$. Then for $f(z,w)=\varphi (w)$ and $g(z,w)=\psi (w)$, we have $fg = 0$ on $\overline{\mathbb {D}^2}$ but $T_{f}T_{g}$ is not compact on $A^2(\mathbb {D}^2)$ as $T_{\varphi }T_{\psi }\not \equiv 0$. This example shows that the vanishing of *fg* on $b\mathbb {D}^2$ (or even on $\overline{\mathbb {D}^2}$) does not imply the compactness of $T_{f}T_{g}$.

### Example 2

Take *f* as in Example 1 and define

$$\begin{aligned} g(z,w) = \varphi (z) + \psi (w). \end{aligned}$$

Then *fg* is not identically zero on $\mathbb {D}^2$ because $f(0,0) = g(0,0) = 1$ and $fg = 0$ on $b\mathbb {D}^2$. Yet, by Theorem 1, the product $T_fT_g$ is not compact since for $\xi \in \mathbb {T}$,

$$\begin{aligned} T_{R_{1,\xi }f}T_{R_{1,\xi }g} = T_{\varphi }T_{\psi } \end{aligned}$$

is not the zero operator on $A^2(\mathbb {D})$.

### Remark 1

From the previous examples we see that $fg=0$ on $b\mathbb {D}^2$ is not a sufficient condition for the compactness of $T_fT_g$. Is it a necessary condition? It turns out this question is related to the zero product problem for Toeplitz operators on the disc. More specifically, as in Example 1, we see that with $f(z,w)=\varphi (w)$ and $g(z,w) = \psi (w)$, if the product $T_fT_g$ is compact on $A^2(\mathbb {D}^2)$, then $T_{\varphi }T_{\psi } = 0$ on $A^2(\mathbb {D})$ (which gives $\varphi \psi = 0$ on $\mathbb {T}$). However, it is not known if this condition implies that $\varphi \psi = 0$ on $\mathbb {D}$. For $\xi \in \mathbb {T}$ and $z, w\in \mathbb {D}$, we have $f(\xi ,w)g(\xi ,w) = \varphi (w)\psi (w)$ and $f(z,\xi )g(z,\xi ) = \varphi (\xi )\psi (\xi )$. So $fg = 0$ on $b\mathbb {D}^2$ if and only if $\varphi \psi = 0$ on $\mathbb {D}$.

In Proposition 1 below, we show that if the symbols are harmonic along the discs in the boundary, then we have necessary and sufficient conditions for the compactness of the product of two Toeplitz operators. A function $f \in C^2(\mathbb {D}^n)$ is said to be *n*-harmonic if

$$\begin{aligned} \Delta _j f = 4\dfrac{\partial ^2 f}{\partial z_j \partial \overline{z}_j}=0, \end{aligned}$$

for all $j=1,2,\dots ,n.$ That is, *f* is harmonic in each variable separately [12, pg. 16].

### Proposition 1

Let $f,g\in C(\overline{\mathbb {D}^n})$ (with $n\ge 2$) such that for $\xi \in \mathbb {T}$, and $1\le k\le n$, the functions $R_{k,\xi }f$ and $R_{k,\xi }g$ are $(n-1)$-harmonic on $\mathbb {D}^{n-1}$. Then $T_f T_g$ is compact if and only if $fg = 0$ on $b\mathbb {D}^n$.

We note that in Example 1, both *f* and *g* depend on the same single variable. In Proposition 2 below, we give a characterization when the symbols are product of single-variable functions.

### Proposition 2

Let $T=\prod ^M_{k=1}T_{f_k}$ be a finite product of Toeplitz operators on $A^2(\mathbb {D}^n)$ such that $f_k(z)=\prod ^{n}_{j=1}f_{j,k}(z_j)$ for $f_{j,k} \in C(\overline{\mathbb {D}})$ and $z=(z_1,\ldots ,z_n)\in \mathbb {D}^n$. Let $F=\prod ^M_{k=1} f_k$. Then the following statements hold.

(i) If *T* is a nonzero compact operator, then $F = 0$ on $b\mathbb {D}^n$.
(ii) If $F = 0$ on $b\mathbb {D}^n$ and *F* is not identically zero on $\mathbb {D}^n$, then *T* is compact.

### Remark 2

We do not know whether (i) in Proposition 2 still holds in the case *T* is the zero operator. This is closely related to the zero product problem. More specifically, consider $f(z,w)=\varphi (w)$ and $g(z,w) = \psi (w)$, where $\varphi , \psi \in C(\overline{\mathbb {D}})$. Then $T = T_fT_g = 0$ on $A^2(\mathbb {D}^2)$ if and only if $T_{\varphi }T_{\psi } = 0$ on $A^2(\mathbb {D})$. On the other hand, $F = fg = 0$ on $b\mathbb {D}^n$ if and only if $\varphi \psi = 0$ on $\mathbb {D}$. It is still an open problem whether $T_{\varphi }T_{\psi } = 0$ on $A^2(\mathbb {D})$ implies that $\varphi \psi = 0$ on $\mathbb {D}$.

### Remark 3

The conclusion of (ii) in Proposition 2 does not hold if *F* is identically zero on $\mathbb {D}^n$. Indeed, the functions *f* and *g* in Example 1 are of the type considered here and $F = fg = 0$ on $\overline{\mathbb {D}^2}$ but $T_{f}T_{g}$ is not compact on $A^2(\mathbb {D}^2)$.

In the proposition below, we show that when all but at most one of the symbols are polynomials, compactness of a Toeplitz product on $A^2(\mathbb {D}^2)$ is equivalent to the vanishing of the product of the symbols on $b\mathbb {D}^2$. For this result, we need to restrict to dimension two. It would be interesting to extend the result to all $n\ge 2$. See Remark 4.

### Proposition 3

Let $f_1,\ldots ,f_M$ and $g_1,\ldots ,g_N$ be polynomials in *z*, *w* and $\overline{z},\overline{w}$, and $h\in C(\overline{\mathbb {D}^2})$. Then $T_{f_1}\cdots T_{f_M}T_hT_{g_1}\cdots T_{g_N}$ is compact on $A^2(\mathbb {D}^2)$ if and only if

$$\begin{aligned} f_1\cdots f_M h g_1\cdots g_N = 0 \text { on } b\mathbb {D}^2. \end{aligned}$$

## Proofs

Let *BT*(*p*) denote the Berezin transform of a bounded linear operator $T:A^2(\mathbb {D}^n)\rightarrow A^2(\mathbb {D}^n)$ at $p\in \mathbb {D}^n$. That is,

$$\begin{aligned} BT(p)=\langle Tk_p,k_p\rangle \end{aligned}$$

where

$$\begin{aligned} k_p(z)=\frac{K(z,p)}{\sqrt{K(p,p)}} \end{aligned}$$

is the normalized Bergman kernel of $\mathbb {D}^n$.

We will need the following lemma whose proof is contained in the proof of Theorem 1 in [13]. We provide a sketch of the proof here for the convenience of the reader. We note that *Bf* denotes $BT_f$ whenever *f* is a bounded function and we use the following notation: $z' =(z_2,\ldots ,z_n)\in \mathbb {C}^{n-1}$ for $z=(z_1,\ldots ,z_n)\in \mathbb {C}^n$. For functions $h_1$ defined on $\mathbb {D}$ and $h_2$ defined on $\mathbb {D}^{n-1}$, we use $h_1h_2$ to denote the function $h_1(z_1)h_2(z')$ on $\mathbb {D}^n$.

### Lemma 1

Suppose $n\ge 2$ and $\psi \in C(\overline{\mathbb {D}^n})$. Let $q=(\zeta ,q')\in \mathbb {T}\times \overline{\mathbb {D}^{n-1}}$ and define $\psi _{\zeta }(z) = \psi (\zeta ,z')$ for $z\in \mathbb {D}^n$.

(i) If $\{h_p: p\in \mathbb {D}^{n}\}$ is a bounded set in $L^2(\mathbb {D}^{n-1})$, then
  $$\begin{aligned} \lim _{p\rightarrow q}\big \Vert (\psi - \psi _{\zeta })k_{p_1}^{\mathbb {D}}h_p\big \Vert = 0. \end{aligned}$$
(ii) If $\psi _1,\ldots ,\psi _v\in C(\overline{\mathbb {D}^{n}})$ are functions independent of $z_1$ and *W* is any bounded operator on $L^2(\mathbb {D}^{n})$, then
  $$\begin{aligned} \lim _{p\rightarrow q}\big \Vert WT_{\psi -\psi _{\zeta }}T_{\psi _1}\cdots T_{\psi _v}k_p\big \Vert = 0. \end{aligned}$$

### Proof

(i) Let $\epsilon >0$ be given. By the uniform continuity of $\psi$, there exists $\delta > 0$ such that for all $z'\in \mathbb {D}^{n-1}$,
  $$|\psi (z_1,z')-\psi _{\xi }(z_1,z')|< \dfrac{\epsilon }{\sup \{\Vert h_p\Vert _{L^2(\mathbb {D}^{n-1})}\}+1} \text { whenever }\ |z_1 - \xi | < \delta . $$
  Then,
  $$\begin{aligned} \Vert (\psi - \psi _{\xi })k^{\mathbb {D}}_{p_1}h_p\Vert ^2&= \Vert (\psi - \psi _{\xi })k^{\mathbb {D}}_{p_1}h_p\Vert ^2_{L^2( \{z \in \mathbb {D}^n:|z_1 - \xi | < \delta \})}\\&\qquad \qquad + \Vert (\psi - \psi _{\xi })k^{\mathbb {D}}_{p_1}h_p\Vert ^2_{L^2( \{z \in \mathbb {D}^n: |z_1 - \xi | \ge \delta \})} \\&\le \epsilon ^2 + \pi \Vert h_p\Vert ^2_{L^2(\mathbb {D}^{n-1})}\Vert (\psi - \psi _{\xi })k^{\mathbb {D}}_{p_1}\Vert ^2_{L^{\infty }(\{z \in \mathbb {D}^n:|z_1 - \xi | \ge \delta \})}. \end{aligned}$$
  However,
  $$\begin{aligned} \sup \left\{ \left| k^{\mathbb {D}}_{p_1}(z_1)\right| : |z_1 - \xi | \ge \delta \right\} \rightarrow 0\ \text {as}\ p_1 \rightarrow \xi . \end{aligned}$$
  Then, $\limsup _{p \rightarrow q}\Vert (\psi -\psi _{\xi })k^{\mathbb {D}}_{p_1}h_p\Vert \le \epsilon$. Since $\epsilon >0$ was arbitrary, we conclude that
  $$\begin{aligned} \lim _{p\rightarrow q}\big \Vert (\psi - \psi _{\zeta })k_{p_1}^{\mathbb {D}}h_p\big \Vert = 0. \end{aligned}$$
(ii) We note that $k_p = k_{p_1}^{\mathbb {D}}k_{p'}^{\mathbb {D}^{n-1}}$ for $p = (p_1,p')$. We define
  $$\begin{aligned} h_p = T_{\psi _1}\cdots T_{\psi _v}k_{p'}^{\mathbb {D}^{n-1}}\text { for } p\in \mathbb {D}^{n}. \end{aligned}$$
  Since each $\psi _j$ is independent of $z_1$, $h_p$ is independent of $z_1$ and hence it can be considered as an element of $L^2(\mathbb {D}^{n-1})$. Note that the set $\{h_p: p\in \mathbb {D}^{n}\}$ is bounded by $\Vert T_{\psi _1}\cdots T_{\psi _v}\Vert$. Furthermore, we have $T_{\psi _1}\cdots T_{\psi _v}k_p = k_{p_1}^{\mathbb {D}}h_p$. It follows that
  $$\big \Vert WT_{\psi -\psi _{\zeta }}T_{\psi _1}\cdots T_{\psi _v}k_p\big \Vert \le \Vert W\Vert \cdot \big \Vert (\psi -\psi _{\zeta })k_{p_1}^{\mathbb {D}}h_p\big \Vert ,$$
  which, by (i), converges to zero as $p\rightarrow q$.

$$\square $$

### Proof of Theorem 1

We first make an observation. If $\varphi$ is a bounded function on $\mathbb {D}^{n-1}$, then $T_{\varphi }$, while initially defined on $A^2(\mathbb {D}^{n-1})$, can be naturally considered as a Toeplitz operator with symbol $E_1\varphi (z_1,z') =\varphi (z')$ acting on $A^2(\mathbb {D}^n)$. This will not create any confusion due to the fact that for $h\in A^2(\mathbb {D}^n)$ independent of $z_1$, the function $T_{E_1\varphi }h$ is also independent of $z_1$ and $(T_{E_1\varphi }h)(z)=(T_{\varphi }h)(z')$ for all $z=(z_1,z')\in \mathbb {D}^{n}$.

Let $\xi \in \mathbb {T}$. For each *j* and $m_j$, the function $f_{j,m_j}$ can be written as $f_{j,m_j} = (f_{j,m_j} -R_{1,\xi }f_{j,m_j})+R_{1,\xi }f_{j,m_j}$. We then expand $T=\sum ^{N}_{j=1} T_{f_{j,1}} \cdots T_{f_{j,m_j}}$ as

$$\begin{aligned} T&= \sum ^{N}_{j=1} \left( T_{R_{1,\xi }f_{j,1}} \cdots T_{R_{1,\xi }f_{j,m_j}} + T_{f_{j,1} -R_{1,\xi }f_{j,1}}T_{R_{1,\xi }f_{j,2}} \cdots T_{R_{1,\xi }f_{j,m_j}} + T_{f_{j,1}}\right. \\&\quad \left. \cdot T_{f_{j,2} - R_{1,\xi }f_{j,2}} T_{R_{1,\xi }f_{j,3}} \cdots T_{R_{1,\xi }f_{j,m_j}} +\cdots +T_{f_{j,1}}T_{f_{j,2}} \cdots T_{f_{j,m_j-1}}T_{f_{j,m_j}-R_{1,\xi }f_{j,m_j}}\right) \\&= \sum ^{N}_{j=1} T_{R_{1,\xi }f_{j,1}} \cdots T_{R_{1,\xi }f_{j,m_j}} + \sum ^{N}_{j=1}\left( T_{f_{j,1} -R_{1,\xi }f_{j,1}}T_{R_{1,\xi }f_{j,2}} \cdots T_{R_{1,\xi }f_{j,m_j}} + T_{f_{j,1}}\right. \\&\quad \left. \cdot T_{f_{j,2}- R_{1,\xi }f_{j,2}}T_{R_{1,\xi }f_{j,3}} \cdots T_{R_{1,\xi }f_{j,m_j}} +\cdots +T_{f_{j,1}}T_{f_{j,2}} \cdots T_{f_{j,m_j-1}}T_{f_{j,m_j}-R_{1,\xi }f_{j,m_j}}\right) . \end{aligned}$$

Note that in the second sum, each summand has the form considered in Lemma 1(ii). We then conclude that for any $q=(\xi ,q')\in \mathbb {T}\times \overline{\mathbb {D}^{n-1}}$,

1 $$\begin{aligned} \lim _{p\rightarrow q}\Big \Vert Tk_p - \sum ^{N}_{j=1} T_{R_{1,\xi }f_{j,1}} \cdots T_{R_{1,\xi }f_{j,m_j}}k_p\Big \Vert = 0. \end{aligned}$$

Now suppose that *T* is compact. Fix $p'\in \mathbb {D}^{n-1}$. Since $k_{(p_1,p')}\rightarrow 0$ weakly as $p_1\rightarrow \xi$, the compactness of *T* implies that $\Vert Tk_{(p_1,p')}\Vert \rightarrow 0$ as $p_1 \rightarrow \xi$. Equation (1) then gives

2 $$\begin{aligned} \lim _{p_1\rightarrow \xi }\Big \Vert \sum ^{N}_{j=1} T_{R_{1,\xi }f_{j,1}} \cdots T_{R_{1,\xi }f_{j,m_j}}k_{(p_1,p')}\Big \Vert = 0. \end{aligned}$$

Since

$$\begin{aligned} \sum ^{N}_{j=1} T_{R_{1,\xi }f_{j,1}} \cdots T_{R_{1,\xi }f_{j,m_j}}k_{(p_1,p')}&= \sum ^{N}_{j=1} T_{R_{1,\xi }f_{j,1}} \cdots T_{R_{1,\xi }f_{j,m_j}}(k_{p_1}^{\mathbb {D}}k_{p'}^{\mathbb {D}^{n-1}}) \\&= k^{\mathbb {D}}_{p_1}\cdot \sum ^{N}_{j=1} T_{R_{1,\xi }f_{j,1}} \cdots T_{R_{1,\xi }f_{j,m_j}}k^{\mathbb {D}^{n-1}}_{p'} \end{aligned}$$

and $\Vert k_{p_1}^{\mathbb {D}}\Vert =1$ for all $p_1$, (2) implies that

$$\sum ^{N}_{j=1} T_{R_{1,\xi }f_{j,1}} \cdots T_{R_{1,\xi }f_{j,m_j}}k^{\mathbb {D}^{n-1}}_{p'} = 0.$$

Because $p'$ was arbitrary, it follows that $\sum ^{N}_{j=1} T_{R_{1,\xi }f_{j,1}} \cdots T_{R_{1,\xi }f_{j,m_j}}$ is the zero operator on $A^2(\mathbb {D}^{n-1})$. Applying the same method for other values of *k*, we have

$$\begin{aligned} \sum ^{N}_{j=1} T_{R_{k,\xi }f_{j,1}} \cdots T_{R_{k,\xi }f_{j,m_j}} = 0 \end{aligned}$$

on $A^2(\mathbb {D}^{n-1})$ for $1 \le k\le n$ and all $\xi \in \mathbb {T}$.

We now prove the converse. Let $q=(\xi ,q') \in b\mathbb {D}^n$ with $\xi \in \mathbb {T}$ and $q'\in \overline{\mathbb {D}^{n-1}}$. Since it is assumed that $\sum ^{N}_{j=1} T_{R_{1,\xi }f_{j,1}} \cdots T_{R_{1,\xi }f_{j,m_j}} = 0$, equation (1) implies that $\lim _{p\rightarrow q}\Vert Tk_p\Vert = 0$. As a consequence,

$$\begin{aligned} \lim _{p\rightarrow q}BT(p) = \lim _{p\rightarrow q}\langle Tk_p,k_p\rangle = 0. \end{aligned}$$

The same argument is applicable for all $q\in b\mathbb {D}^{n}$. By Axler–Zheng Theorem for $\mathbb {D}^n$ ([2] and [3, p. 232]), we conclude that *T* is compact on $A^2(\mathbb {D}^n)$.

### Proof of Corollary 1

We assume that for each $\xi \in \mathbb {T}$ and $1\le k \le n$ there exists *j* such that $R_{k,\xi }f_j=0$. Then $T_{R_{k,\xi }f_m}\cdots T_{R_{k,\xi }f_{1}}=0$ on $A^2(\mathbb {D}^{n-1})$. Hence, Theorem 1 implies that $T_{f_m}\cdots T_{f_1}$ is compact on $A^2(\mathbb {D}^n)$.

### Proof of Proposition 1

To prove the forward direction, we first use Theorem 1 to conclude that the operator $T_{R_{k,\xi }g}T_{R_{k,\xi }f}$ is zero on $A^2(\mathbb {D}^{n-1})$ for all $\xi \in \mathbb {T}$ and $1\le k \le n$. Since the symbols $R_{k,\xi }f$ and $R_{k,\xi }g$ are $(n-1)$-harmonic on $\mathbb {D}^{n-1}$, we apply [14, Theorem 1.1] (or [15, Corollary 2] in the case $n=2$) to conclude that either $R_{k,\xi }f=0$ or $R_{k,\xi }g=0$. Then $fg=0$ on $b\mathbb {D}^n$ as desired.

To prove the converse we argue as follows. For each $1\le k\le n$ and $\xi \in \mathbb {T}$, since both $R_{k,\xi }f$ and $R_{k,\xi }g$ are $(n-1)$-harmonic and their product is zero on $\mathbb {D}^{n-1}$, either $R_{k,\xi }f=0$ or $R_{k,\xi }g=0$. Then $T_{R_{k,\xi }g}T_{R_{k,\xi }f}=0$ on $A^2(\mathbb {D}^{n-1})$ for all $\xi \in \mathbb {T}$ and $1 \le k \le n$. Theorem 1 now implies that $T_gT_f$ is compact.

### Proof of Proposition 2

We first prove (i). Assume that *T* is a nonzero compact operator. Then by Theorem 1 when restricted on the first coordinate, for any $\xi \in \mathbb {T}$,

$$0 = \prod _{k=1}^{M}T_{R_{1,\xi }f_k} =\Big (\prod _{k=1}^{M}f_{1,k}(\xi )\Big )\prod _{k=1}^{M}T_{\widetilde{f}_k}$$

on $A^2(\mathbb {D}^{n-1})$, where $\widetilde{f}_k(z_2,\ldots ,z_n) = f_{2,k}(z_2)\cdots f_{n,k}(z_n)$. Since *T* is not the zero operator, the second factor on the right hand side above is a nonzero operator. This follows from the fact that *T* can be written as the product

$$\displaystyle \big (\prod _{k=1}^{M}T_{f_{1,k}}\big ) \cdot \big (\prod _{k=1}^{M}T_{\widetilde{f}_k}\big )$$

where the first factor acts on functions in $z_1$ and the second factor acts on functions in $z' = (z_2,\ldots ,z_n)$. Hence, $\prod _{k=1}^{M}f_{1,k}(\xi )=0$. It follows that

$$F(\xi ,z_2,\dots ,z_n)=\prod _{k=1}^{M}f_k(\xi ,z_2,\dots ,z_n) =\left( \prod _{k=1}^{M}f_{1,k}(\xi )\right) \left( \prod ^{n}_{j=2} \prod _{k=1}^{M}f_{j,k}(z_j)\right) =0$$

on $\mathbb {T}\times \mathbb {D}^{n-1}$. The same argument applies to other coordinates and we have $F = 0$ on $b\mathbb {D}^n$.

Next we prove (ii). Assume that $F=\prod ^M_{k=1} f_k = 0$ on $b\mathbb {D}^n$ and *F* is not identically zero on $\mathbb {D}^n$. Choose $q=(q_1,\ldots ,q_n)\in \mathbb {D}^n$ such that $f_k(q)\ne 0$ for all *k*, which implies that $f_{j,k}(q_j)\ne 0$ for all *j* and *k*. For any $\xi \in \mathbb {T}$, since $z=(\xi ,q_2,\ldots ,q_n)\in b\mathbb {D}^n$, we have

$$0 = F(z) = \Big (\prod _{k=1}^{M}f_{1,k}(\xi )\Big ) \cdot \prod _{j=2}^{n}\prod _{k=1}^{M}f_{j,k}(q_j).$$

Because the second factor is nonzero, it follows that $\prod _{k=1}^{M}f_{1,k}(\xi ) = 0$. As a result,

$$\prod _{k=1}^{M}T_{R_{1,\xi }f_k} = \Big (\prod _{k=1}^{M}f_{1,k}(\xi )\Big )\prod _{k=1}^{M}T_{\widetilde{f}_k} = 0$$

on $A^2(\mathbb {D}^{n-1})$, where, as before, $\widetilde{f}_k(z_2,\ldots ,z_n) = f_{2,k}(z_2)\cdots f_{n,k}(z_n)$. The same argument applies to other parts of $b\mathbb {D}^n$. Then Theorem 1 implies that $T=\prod _{k=1}^{M}T_{f_k}$ is compact on $A^2(\mathbb {D}^n)$.

The proof of Proposition 3 hinges on several elementary facts about polynomials that we describe below. We use $\mathbb {C}[z,\overline{z}]$ to denote the vector space of all polynomials in *z* and $\overline{z}$.

The following lemma is well known. The proof follows from the fact that if a real analytic function vanishes on a non-empty open set, it must be identically zero.

### Lemma 2

Let $f\in \mathbb {C}[z,\overline{z}]$ be not identically zero. Then the set

$$\begin{aligned} \{z\in \mathbb {C}: f(z) = 0\} \end{aligned}$$

has an empty interior.

### Lemma 3

Let $f\in \mathbb {C}[z,\overline{z}]$. Assume that there exist infinitely many $\xi \in \mathbb {T}$ such that $f(\xi )=0$. Then there is a polynomial $g\in \mathbb {C}[z,\overline{z}]$ such that $f(z) = (1-|z|^2)g(z)$. In particular, $f(\xi )=0$ for all $\xi \in \mathbb {T}$.

### Proof

For non-negative integers *s*, *t*, we write

$$\overline{z}^s z^t = {\left\{ \begin{array}{ll} |z|^{2\,s}\,z^{t-s} &  \text { if } t\ge s,\\ |z|^{2t}\,\overline{z}^{s-t} &  \text { if } t < s. \end{array}\right. }$$

As a result, there are integers $m,M\ge 0$ and polynomials $p_j$ (for $0\le j\le M$) and $q_j$ (for $0\le j\le m$) of a single variable such that

$$\begin{aligned} f(z) = \sum _{j=0}^{M}p_j(|z|^2)z^{j} + \sum _{j=0}^{m}q_j(|z|^2)\overline{z}^{j}. \end{aligned}$$

By the hypothesis, there exists infinitely many $\xi \in \mathbb {T}$ such that

$$\begin{aligned} \sum _{j=0}^{M}p_j(1)\xi ^{j} + \sum _{j=0}^{m}q_j(1)\overline{\xi }^j = f(\xi ) = 0. \end{aligned}$$

This implies that $p_j(1) = q_j(1) = 0$ for each *j*. As a consequence, all $p_j(r)$ and $q_j(r)$ are divisible by $1-r$. We then conclude that *f*(*z*) is divisible by $1-|z|^2$, from which the conclusion of the lemma follows.

### Lemma 4

Let *f*(*z*, *w*) be a polynomial in $z,w,\overline{z},\overline{w}$ and let $h \in C(\overline{\mathbb {D}^2})$. Assume that $fh = 0$ on $b\mathbb {D}^2$. Then $f|_{\mathbb {T}\times \overline{\mathbb {D}}}=0$ or $h|_{\mathbb {T}\times \overline{\mathbb {D}}}=0$ and $f|_{\overline{\mathbb {D}}\times \mathbb {T}}=0$ or $h|_{\overline{\mathbb {D}}\times \mathbb {T}}=0$.

### Proof

Assume that *h* does not vanish identically on $\mathbb {T}\times \overline{\mathbb {D}}$. By continuity, there exist a non-empty arc $J\subseteq \mathbb {T}$ and a non-empty open set $V\subseteq \mathbb {D}$ such that $h(\xi ,w)\ne 0$ for all $\xi \in J$ and $w\in V$. It follows that $f(\xi ,w)=0$ for all such $\xi$ and *w*. For each $\xi \in J$, applying Lemma 2, we conclude that $f(\xi ,w)=0$ for all $w\in \overline{\mathbb {D}}$. Then for each $w\in \overline{\mathbb {D}}$, since $f(\xi ,w)$ vanishes on *J* (which is an infinite set), Lemma 3 implies that $f(\xi ,w)=0$ for all $\xi \in \mathbb {T}$. Therefore, *f* vanishes identically on $\mathbb {T}\times \overline{\mathbb {D}}$. The proof for $\overline{\mathbb {D}}\times \mathbb {T}$ is similar.

### Lemma 5

[[16, Corollary 1.8]] Suppose $\varphi _1,\ldots ,\varphi _M$ and $\psi _1,\ldots ,\psi _N$ are polynomials of $z, \overline{z}$ in $\mathbb {D}$ and $g\in L^{2}(\mathbb {D})$. If $T_{\varphi _1}\cdots T_{\varphi _M}T_{g}T_{\psi _1}\cdots T_{\psi _N}=0$ on $A^2(\mathbb {D})$, then one of the symbols must be zero.

### Proof of Proposition 3

Assume that $T_{f_1}\cdots T_{f_M}T_hT_{g_1}\cdots T_{g_N}$ is compact on $A^2(\mathbb {D}^2)$, then by Theorem 1,

$$ T_{R_{1,\xi }f_1}\cdots T_{R_{1,\xi }f_M}T_{R_{1,\xi }h}T_{R_{1,\xi }g_1} \cdots T_{R_{1,\xi }g_N} = 0$$

on $A^2(\mathbb {D})$ for all $\xi \in \mathbb {T}$. By Lemma 5, one of $R_{1,\xi }f_1,\ldots , R_{1,\xi }f_M$, $R_{1,\xi }h$, and $R_{1,\xi }g_1,\ldots ,R_{1,\xi }g_N$ is a zero function on $\mathbb {D}$. Thus, $f_1\cdots f_M h g_1\cdots g_N = 0$ on $\mathbb {T}\times \overline{\mathbb {D}}$. Similar argument works for $\overline{\mathbb {D}} \times \mathbb {T}$. Therefore, $f_1\cdots f_M h g_1\cdots g_N = 0$ on $b\mathbb {D}^2$.

For the converse, by Lemma 4, one of the symbols is identically zero on $\mathbb {T}\times \overline{\mathbb {D}}$. It then follows that

$$T_{R_{1,\xi }f_1}\cdots T_{R_{1,\xi }f_M}T_{R_{1,\xi }h}T_{R_{1,\xi }g_1} \cdots T_{R_{1,\xi }g_N}=0.$$

Similarly,

$$T_{R_{2,\xi }f_1}\cdots T_{R_{2,\xi }f_M}T_{R_{2,\xi }h}T_{R_{2,\xi }g_1} \cdots T_{R_{2,\xi }g_N}=0.$$

Therefore, by Theorem 1, we conclude that $T_{f_1}\cdots T_{f_M}T_hT_{g_1}\cdots T_{g_N}$ is compact on $A^2(\mathbb {D}^2)$.

### Remark 4

It is desirable to generalize Proposition 3 to $\mathbb {D}^n$ for all $n\ge 2$. While Lemmas 2, 3 and 4 remain true for all *n*, Lemma 5 has only been known for the disc. In order to extend Proposition 3 to all $n\ge 2$, one needs to prove a several-variable version of Lemma 5. Some partial results have been obtained in the literature. For example, the main results of [17] imply that Lemma 5 holds in several variables when $g=1$ or when all $\varphi _j, \psi _k$ are monomials. As a result, Proposition 3 holds on $\mathbb {D}^n$ for all $n\ge 2$ in the case $h=1$, or in the case all $f_j$ and $g_k$ are monomials.

## Data Availability

Data sharing is not applicable to this article as no data sets were generated or analyzed in this study.
